# PRISMA flow diagrams for living systematic reviews: a methodological survey and a proposal

**DOI:** 10.12688/f1000research.51723.1

**Published:** 2021-03-08

**Authors:** Lara A. Kahale, Rayane Elkhoury, Ibrahim El Mikati, Hector Pardo-Hernandez, Assem M. Khamis, Holger J. Schünemann, Neal R. Haddaway, Elie A. Akl

**Affiliations:** 1 Clinical Research Institute, Cochrane, London, Beirut, Riad El Solh 1107 2020, UK; 2 Faculty of Medicine, Weill Cornell Medicine-Qatar, Cornell University, Beirut, Riad El Solh 1107 2020, Qatar; 3 Iberoamerican Cochrane Centre, Sexually Transmitted Infections, and Viral Hepatitis, Weill Cornell Medicine–Qatar, Barcelona, C / Sant Quintí, 77-79 08041, Qatar; 4 American University of Beirut, Madrid, Av. de Monforte de Lemos, 5, 28029, Lebanon; 5 Wolfson Palliative Care Research Centre, Hull York Medical School, Sant Pau Biomedical Research Institute, Hull, Hull HU6 7RX, Spain; 6 Department of Health Research Methods, Evidence, and Impact, CIBER Epidemiología y Salud Pública, Hamilton, Ontario, 1280 Main Street West 2C Area, Spain; 7 Department of Medicine, Hull York Medical School, University of Hull, Hamilton, Ontario, 1280 Main Street West 2C Area, UK; 8 McMaster University, Stockholm, Linnégatan 87D, Canada; 9 McMaster University, Berlin, Torgauer Strasse 19, Canada; 10 Africa Centre for Evidence, Stockholm Environment Institute, Johannesburg, Sweden; 11 Department of Internal Medicine, Leibniz Centre for Agricultural Landscape Research (ZALF), Beirut, Riad El Solh 1107 2020, Germany

**Keywords:** PRISMA statement, living systematic review, update, research methodology research reporting, flow chart, systematic review reporting standards, evidence synthesis, research transparency, research replication

## Abstract

**Background**: While the PRISMA flow diagram is widely used for reporting standard systematic reviews (SRs), it was not designed for capturing the results of continual searches for studies in living systematic reviews (LSRs). The objectives of this study are (1) to assess how published LSRs report on the flow of studies through the different phases of the review for the different updates; (2) to propose an approach to reporting on that flow.

**Methods**: For objective 1, we identified all LSRs published up to July 2020. We abstracted information regarding their general characteristics and how they reported on search results. For objective 2, we based our proposal for tailored PRISMA approaches on the findings from objective 1, as well as on our experience with conducting Cochrane LSRs.

**Results: **We identified 108 living publications relating to 32 LSRs. Of the 108 publications, 7% were protocols, 24% were base versions (i.e., the first version), 62% were partial updates (i.e., does not include all typical sections of an SR), and 7% were full updates (i.e., includes all typical sections of an SR). We identified six ways to reporting the study flow: base separately, each update separately (38%); numbers not reported (32%); latest update separately, all previous versions combined (20%); base separately, all updates combined (7%); latest update version only (3%);  all versions combined (0%). We propose recording in detail the results of the searches to keep track of all identified records. For structuring the flow diagram, we propose using one of four approaches.

**Conclusion:** We identified six ways for reporting the study flowthrough the different phases of the review for the different update versions. We propose to document in detail the study flow for the different search updates and select one of our four tailored PRISMA diagram approaches to present that study flow.

## Introduction

During the coronavirus disease 2019 (COVID-19) pandemic, health research has proliferated exponentially ^1^. Systematic reviews are essential to synthesize the evidence and inform policy and practice. Given the pace of research publication, those reviews need to be kept up to date. Living systematic reviews (LSRs) are an emerging type of systematic review that involves the continual search of the literature and incorporation of relevant new evidence, soon after it becomes available ^2^. While many evidence synthesis groups are engaged in conducting LSRs or living network meta-analyses, others have developed living databases or living maps, including resources specific for COVID-19 literature ^3– 17^.

An essential component of systematic reviews is to keep track of and report the number of records captured while searching the scientific literature and details of the selection process ^18^. The PRISMA statement recommends the use of the PRISMA flow diagram to depict the flow of studies through the different phases of the systematic review ^19^. While the PRISMA flow diagram is a widely used tool for reporting original systematic reviews, it was not designed to capture the results of continual searches typically used in LSRs. Hence, it’s unclear how authors of LSRs address the issue of presenting results of these continual searches.

The objectives of this study were (1) to assess how published LSRs report on the flow of studies through the different phases of the review for the different updates; and (2) to propose an approach to documenting and reporting on the flow of studies through the different phases of a LSR, for the different updates.

## Methods

For objective 1, we collected relevant data as part of a larger methodological survey aiming to assess the methods of conduct and reporting of LSRs. We have described the details of that study in a previously published protocol ^20^. Briefly, we identified all living reviews published up to July 2020 available from the following electronic databases: Medline, EMBASE and the Cochrane library (see extended data ^21^ of Khamis *et al.* ^20^ for the search strategy). An eligible living review was either (1) a protocol for an LSR, (2) a base version of an LSR, (3) a full update version of an LSR, (4) a partial update version of an LSR, or (5) a combination of any of these (e.g., one living review may constitute of a protocol, a base version, and a full update version; another living review may constitute of only a Box 1 the definition of each type of living reviews.

**Box 1. Definition of the different publication types of living reviews**

•  LSR protocol: the protocol that describes the planned methods of the living review

•  Base version: the first version of the review that follows a living approach

•  Full update version: a subsequent version of the review that includes all the typical sections of a systematic review, including an introduction, methods, and results sections. Such a version could stand-alone in terms of content.

•  Partial update version: a subsequent version of the review that does not include all the typical sections of a systematic review, but instead refers to a previous version for complementary information. Such a version could not stand-alone in terms of content.

For the current study, we abstracted information about the following features of LSRs:

- General characteristics:
  - Publication type, i.e., protocol, base version, full update version, partial update version.
  - Whether published in the Cochrane library or elsewhere.
  - Field (e.g., clinical, public health)
  - Whether COVID-19 related or not
  - Whether the base version of the living review conducted as a rapid review or not
- Reporting on study flow
  - Method used to report on the study flow (including the search results and the results of the selection process):
    ▪ Narrative format and/or flow diagram.
    ▪ Whether the results of the base and update searches are reported separately or not.
  - Type of flow diagram, if applicable (e.g., PRISMA).

For objective 2, we base our proposal for tailored PRISMA diagram approaches on the findings from objective 1, on our experience conducting Cochrane LSRs, and our methodological work on designing and reporting living evidence. Since 2017, our group has been responsible for the first series of three Cochrane LSRs, all of which address anticoagulation in patients with cancer ^22– 24^. We conducted the base search in February 2016. Since then, we have been updating the search on a monthly basis. Through this experience, we have been able to apply and refine the guidance for conducting LSRs endorsed by the living evidence network group ^25^. Specifically, we explored solutions for the reporting of the study flow that would address different scenarios. Our goal was not to be prescriptive and narrow, but rather to cover all possible resulting flows by reviewing the LSRs we identified based on objective 1. Two authors developed a draft of the tailored approaches to presenting the study flow, and then circulated to the author team for review and suggestions for improvement.

## Data handling and analysis

We used REDCap to collect and manage the data abstraction process. All data were exported from REDCap and analyzed using Stata v. 13 ^26, 27^.

## Results

### Survey findings

Our search identified a total of 108 living publications relating to 32 LSRs. Table 1 shows their general characteristics. Of the 108 living publications, 8% were protocols, 24% were base versions, 61% were partial updates, and 8% were full updates. The median number of living publications per LSR was 1 (Interquartile range 1–4). Of the 32 living reviews, 31% were published in the Cochrane library, 30% were related to COVID-19, and 15% had a base version published as a rapid review. The majority were related to clinical topics (78%).

**Table 1.** General characteristics of the 108 included living publications related to 32 living reviews.

	N	n (%)
**Publication type**	*108 living publications*	
• Protocol		8 (7.4)
• Base version		26 (24.1)
• Partial update version		66 (61.1)
• Full update version		8 (7.4)
**Living publications per LSR** (Median (IQR)	*32 LSRs*	1 (1 – 4)
**Cochrane LSR**	*32 LSRs*	10 (31.3)
**Field**	*32 LSRs*	
• Clinical		25 (78.1)
• Public health		5 (15.6)
• Health system and policy		2 (6.3)
**COVID-19-related**	*32 LSRs*	10 (30.3)
**Base version published as rapid review**	*26 base versions*	4 (15.4)

***Abbreviations**: LSR: living systematic review; IQR: interquartile range*

Table 2 shows the results for the reporting on the study flow. Most base versions and full updates used a flow diagram to report on the search results (96% and 100% respectively), whereas none of the partial updates presented a flow diagram.

**Table 2.** Reporting on study flow.

	N	n (%)
**Inclusion of a flow diagram in ^a^**		
• Base version	*26 base versions*	25 (96.2)
• Partial update version	*67 partial updates*	1 (1.5)
• Full update version	*7 full updates*	7 (100.0)
**Approach to reporting on study flow for** **different versions**	*74 publications of LSRs that* *included at least one update*	
Base separately; each update separately		28 (37.8)
Numbers not reported		24 (32.4)
Latest update separately; all previous versions combined (including the base)		15 (20.1)
Base separately; all updates combined		5 (6.8)
Latest update version only		2 (2.7)
All versions combined		0

^a^ When a flow diagram is not reported, the authors reported on the search results in a narrative format.

**Figure 1.**
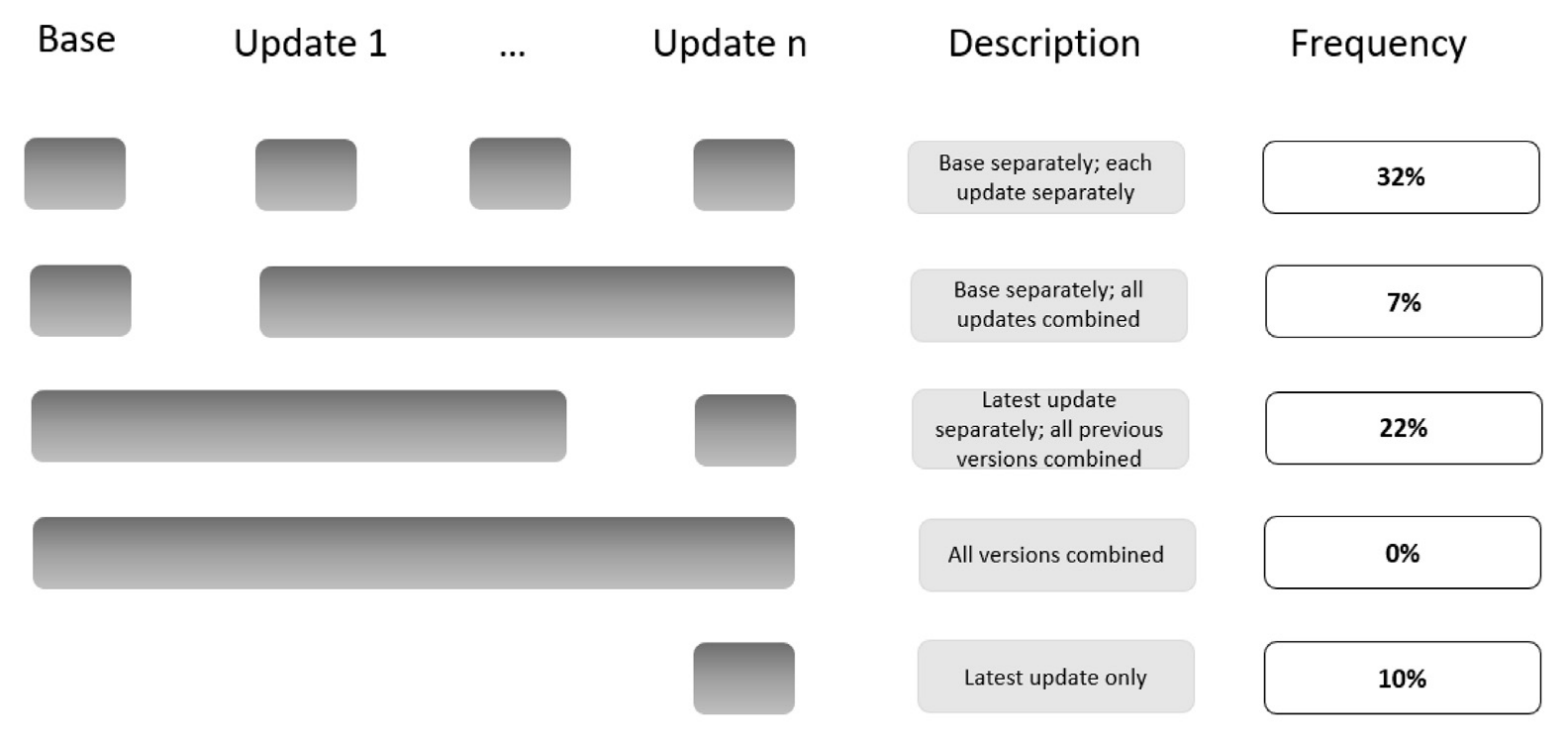
Summary of the four PRISMA tailored approaches to present that study to presenting on the study flow for the different search updates.

Among the 74 publications related to LSRs that included at least one update (12 LSRs) ( Figure 1):

- 38% reported the search results for the base version and for each update version separately.
- 32% did not report the search results at all (e.g., ‘new studies identified and integrated’ without specifying the number).
- 20% reported the search results for the latest update version separately and for all previous versions combined (including the base).
- 3% reported the search results for the latest update version only.
- 7% reported on the search results for the base version separately and for all update versions combined.
- 0% reported the search results for all the different versions combined.

### Proposed tailored PRISMA approaches

Using the approach described in the methods section, we developed four approaches that allow authors to document and report the study flow for the different review update versions of an LSR.

***1. Documenting LSR study flow***

Authors should record in detail the results of the searches to keep track of all identified records. We propose using a spreadsheet for one LSR at a time. The format we present consists of tabs for each of the respective search sources: bibliographic databases (e.g. MEDLINE, EMBASE, Cochrane databases); conference proceedings; ongoing studies as captured in clinicaltrials.gov and WHO International Clinical Trials Registry Platform (ICTRP); other tabs as needed, and a final ‘cumulative’ tab.

**Figure 2.**
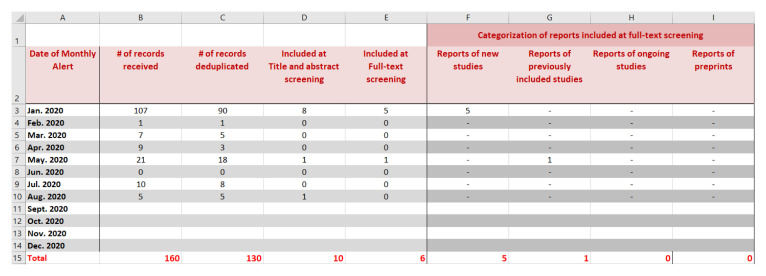
Snapshot of the ‘cumulative’ tab of the spreadsheet that keep track of all identified records.

We show in Figure 2 a snapshot of the ‘cumulative’ tab of the spreadsheet that keeps track of all records. It shows the study flow for a hypothetical example for an LSR published first in January 2020 (i.e. base version) and updated on a monthly basis up to August 2020. Each row corresponds to a different update version. The columns present the following information for each update (columns B to E): the number of records received, deduplicated, included at title and abstract screening, and included at full-text screening (i.e., newly included reports). Additional columns (F to I) present the distribution of the newly included reports as relating to either: (1) new studies, (2) previously included studies, (3) ongoing (unpublished) studies, or (4) preprints.

After manually entering the information in the first five tabs (corresponding to the different search sources) the total is automatically computed in the ‘cumulative’ tab. 

***2. Reporting LSR study flow***

**Figure 3.**
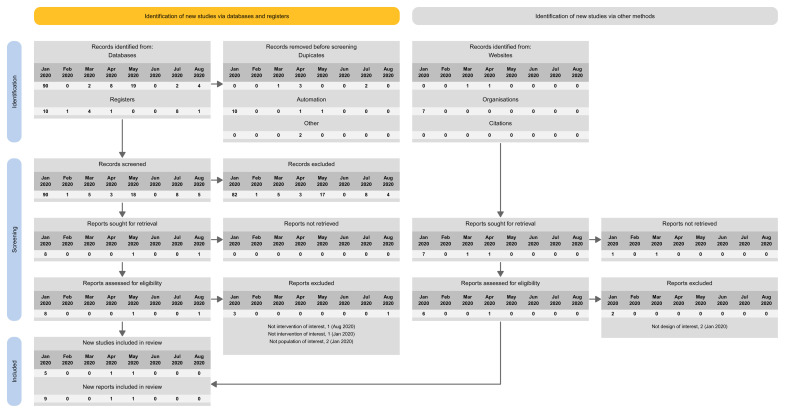
Approach 1: presenting the search results of the different versions separately (i.e., base and each update separately).

**Figure 4.**
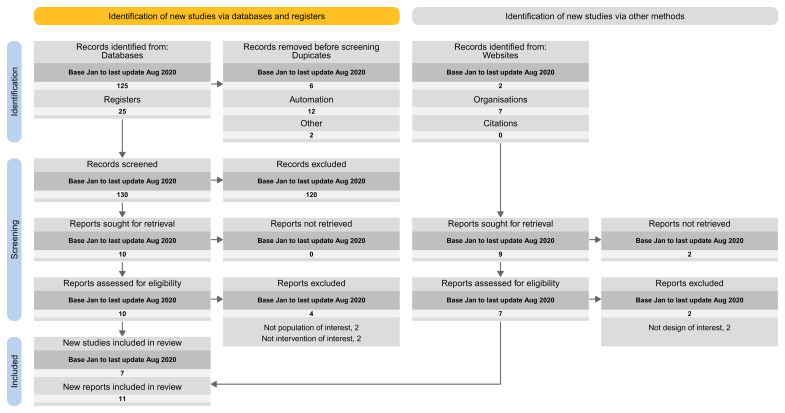
Approach 2: presenting the search results for the different versions combined (i.e., including base and all update versions).

**Figure 5.**
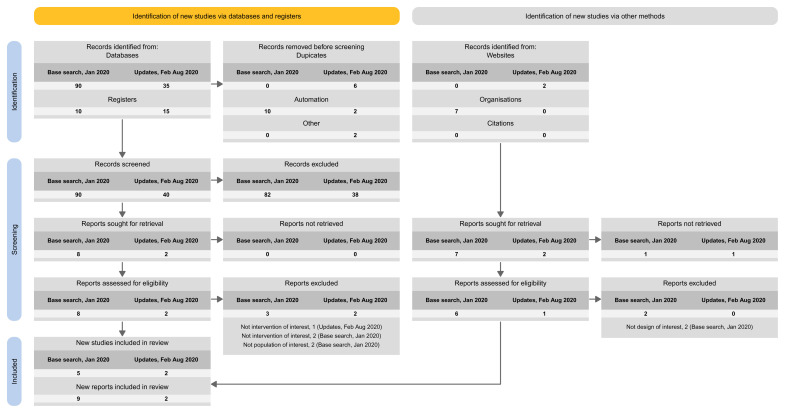
Approach 3: presenting the search results for the base version separately, and the results of all update version combined.

**Figure 6.**
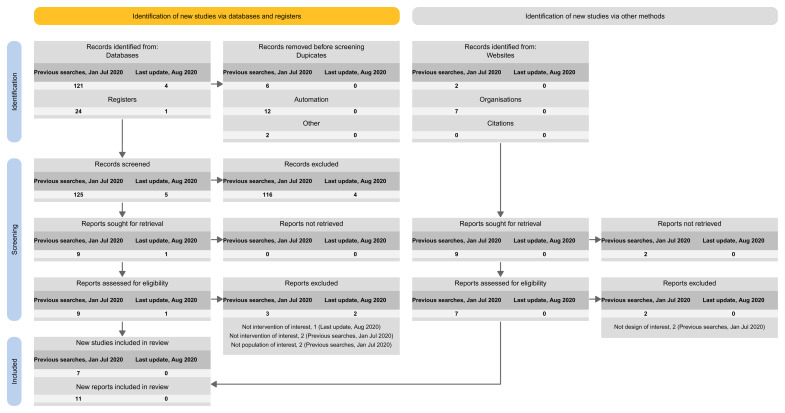
Approach 4: presenting the results of the latest update version separately, and the results of all previous versions (including the base) combined.

The proposed spreadsheet above can act as a basis for a PRISMA flow diagram for LSRs. For structuring the flow diagram for LSR, one can select one out of four tailored PRISMA approaches:

- Approach 1: presenting the search results of the different versions separately (i.e., base and each update separately) ( Figure 3).
- Approach 2: presenting the search results for the different versions combined (i.e., including base and all update versions) ( Figure 4).
- Approach 3: presenting the search results for the base version separately, and the results of all update version combined ( Figure 5).
- Approach 4: presenting the results of the latest update version separately, and the results of all previous versions (including the base) combined ( Figure 6).

In our Cochrane reviews, we applied the second proposal where we present the results for the different searches combined.

## Discussion

### Summary

This study found that authors of LSRs are not consistent in reporting on the flow of studies through the different phases of the review for the different update versions. Thus, we propose to document in detail the study flow for the different search updates and select one of four tailored PRISMA diagram approaches to present that study flow.

### Strengths and limitations

To our knowledge, this is the first methodological survey that assesses how LSR authors report on the flow of studies through the different phases of the review for the different update versions of LSRs. In addition, the research expertise on our team covers both living approach and regular updating of traditional SR. We believe that our assessment forms a vital baseline and allows us to propose best practices for visualization options to improve consistency whilst the production of LSRs is still at a relatively early stage. Indeed, this survey is part of a larger methodological survey aiming to assess the methods of conduct and reporting of LSRs ^20^, that would allow us to update our findings in the future.

### Interpretation of findings

Authors tend more towards producing partial updates of LSRs rather than continually updating the full manuscript in a form of a full update. This might seem like a pragmatic approach particularly for a rapidly growing research field and when methods do not seem to change from one update to another. The heterogeneity observed in the ways LSR authors report on the study flow is likely to be explained by the lack of clear guidance on how to do so.

### Implications for practice

We built our proposal on the PRISMA 2020 flow diagram and provide four approaches to tailor the needs for continual searchers used in LSR. The fourth approach is the closest to the current PRISMA 2020 flow diagram as it presents the results of the latest update version separately and the results of all previous versions (including the base) combined.

In addition, we proposed three other different approaches to provide options to LSR authors and publishing journals. Whatever approach one decides to follow, for transparency purposes, the systematic reviewers should ideally archive previous versions of the flow diagram (e.g., in an appendix). One major challenge will be to accommodate a large number of updates in the same diagram; some approaches would work better than others in that case. Also, advanced information technology solutions may allow fitting a large number of updates, for example as shown in our web-based prototypes that allow ‘toggling’ between approaches. Once an approach is selected, one may develop an interactive flow diagram that have been designed to facilitate developing and communicating flow diagrams concisely ^28^.

Advanced information technology can also be utilized to simplify updating and tracking the change in all LSR sections including the PRISMA diagram. It would be optimal to develop the base version in a certain platform where all SR and LSR sections are reported as units (i.e., title, authors, background, objectives, inclusion criteria, effect estimate for outcome x). With each update and for every unit, the author has the luxury to keep the same text (if no change has occurred) or edit (if change has occurred). Each unit can be updated in a differential speed based on certain criteria. The edits could be highlighted to visualize the change. For a certain section, one would easily have access to the entries in the previous versions and possibly visualize a trend across the different versions (i.e., cross-sectional view for that specific item). For example, dynamic documents can be developed using ‘R markdown’, a document preparation system, where static text can be combined with in-line code and ‘code chunks’ that produce instantly updatable documents given a modified input ^29^.

### Implications for future research

Future research should pilot the proposed approaches for documenting the study flow and for structuring the living flow diagram. In addition, qualitative studies would be helpful to explore: (1) the feasibility and acceptability by LSR authors, publishers, and users towards the proposal; and, (2) what the end-users would like to see in an LSR update.

## Conclusions

LSR authors are not consistent in reporting the flow of studies through the different phases of the review for the different update versions. We propose to document in detail the study flow for the different search updates and select one of our four PRISMA diagram approaches to present that study flow. Finally, improving the reporting of study flow in LSR methodology is essential for incorporating living evidence when developing living guidance, particularly in the context of an urgent response ^30, 31^.

## Data availability

### Underlying data

All data underlying the results are available as part of the article and no additional source data are required.
