# Supplementary material for: Tailored PRISMA 2020 flow diagrams for living systematic reviews: a methodological survey and a proposal
Source: F1000Res. 2022 Jan 28;10:192. Originally published 2021 Mar 8. [Version 3] doi: 10.12688/f1000research.51723.3 (PMC8804909; doi:10.12688/f1000research.51723.3)
Supplement: Peer review history

# Peer review: Reviewer response for version 3

**DOI:** 10.5256/f1000research.120090.r121494

**Published:** 2022-01-31

**Authors:** Lex M. Bouter

**Affiliations:** 1 Department of Epidemiology and Data Science, Amsterdam Universities Medical Center, Amsterdam, The Netherlands; 2 Department of Philosophy, Faculty of Humanities, Vrije Universiteit, Amsterdam, The Netherlands

I applaud the authors for updating their survey and for more than double the sample size. They also made some other - rather marginal -  changes and explain that their publication is only the first of a series based on an ongoing project.

Is the work clearly and accurately presented and does it cite the current literature?

Yes

If applicable, is the statistical analysis and its interpretation appropriate?

No

Are all the source data underlying the results available to ensure full reproducibility?

Yes

Is the study design appropriate and is the work technically sound?

Partly

Are the conclusions drawn adequately supported by the results?

Yes

Are sufficient details of methods and analysis provided to allow replication by others?

Yes

Reviewer Expertise:

Methodology, epidemiology, research integrity, open science, systematic review methods.

I confirm that I have read this submission and believe that I have an appropriate level of expertise to confirm that it is of an acceptable scientific standard.

# Peer review: Reviewer response for version 2

**DOI:** 10.5256/f1000research.55448.r87133

**Published:** 2021-07-22

**Authors:** Sonia Hines

**Affiliations:** 1 College of Medicine and Public Health, Flinders University, Alice Springs, Northern Territory, Australia

This is an interesting proposal to solve the problem of study flow reporting in living systematic reviews (LSRs). As LSRs increase in number, the methodology and reporting requirements need to be well described and usable.

 I am not sure these particular suggested approaches are the most practical. Spreadsheets are not easily inserted into publications, but it is a worthwhile question to be asking, and working from the existing publications is a good starting point.

 The authors have done what they set out to do, but I suggest further work is needed before a recommended method of study flow reporting is settled upon.

Is the work clearly and accurately presented and does it cite the current literature?

Yes

If applicable, is the statistical analysis and its interpretation appropriate?

Yes

Are all the source data underlying the results available to ensure full reproducibility?

Yes

Is the study design appropriate and is the work technically sound?

Yes

Are the conclusions drawn adequately supported by the results?

Yes

Are sufficient details of methods and analysis provided to allow replication by others?

Yes

Reviewer Expertise:

Systematic reviews, evidence-based practice.

I confirm that I have read this submission and believe that I have an appropriate level of expertise to confirm that it is of an acceptable scientific standard.

# Response

**Published:** 2022-01-07

**Authors:** Lara Kahale

**Affiliations:** American University of Beirut, Lebanon

The Reviewers’ comments are in **bold** font and our replies in regular font. Extracts from the text are in *italic* fonts with changes *underlined*. We have indicated the sections where revisions have been made in our manuscript.

Reviewer 2: Sonia Hines

**1. This is an interesting proposal to solve the problem of study flow reporting in living systematic reviews (LSRs). As LSRs increase in number, the methodology and reporting requirements need to be well described and usable. I am not sure these particular suggested approaches are the most practical. Spreadsheets are not easily inserted into publications, but it is a worthwhile question to be asking, and working from the existing publications is a good starting point.**

- Thank you for your positive feedback and practical advice. We agree that spreadsheets are not easily publishable, that is why we recommend using it as a tool for the LSR authors to document in detail the study flow as opposed to using it as a reporting tool. We now elaborate in the discussion how this spreadsheet would feed in an app (shinyapp) that creates nicely designed flows for these four approaches well suited for insertion into a publication.  

**2. The authors have done what they set out to do, but I suggest further work is needed before a recommended method of study flow reporting is settled upon.**

- We agree with this comment. This study is a methodological survey aiming to summarize what LSR authors are currently reporting. As this is not a guidance document, we are not making any recommendations. However, this study is part of a bigger project aiming to develop extension to the PRISMA 2020 statement for LSRs. For that purpose, we will be following the EQUATOR Network’s guidance for developing health research reporting guidelines which include, but is not limited to, engaging stakeholders, including methodologists, LSR end-users, Cochrane groups, journal editors, etc. in a Delphi exercise. In addition, we will be doing a scoping review of current guidance documents and methods papers, and a qualitative study with stakeholders.

# Peer review: Reviewer response for version 2

**DOI:** 10.5256/f1000research.55448.r87481

**Published:** 2021-06-28

**Authors:** Lex M. Bouter

**Affiliations:** 1 Department of Epidemiology and Data Science, Amsterdam Universities Medical Center, Amsterdam, The Netherlands; 2 Department of Philosophy, Faculty of Humanities, Vrije Universiteit, Amsterdam, The Netherlands

Living Systematic Reviews (LSRs) are updated as new evidence becomes available and gained popularity during the Covid-19 pandemic. This manuscript describes the way PRISMA 2020 flow diagrams are handled in 32 LSRs with a view to recommend how this can best be done. The topic is relevant albeit a bit narrow and the manuscript is written clearly. However, there’re some issues that should to be solved in the next version of the manuscript.

**Major issues**

- It’s disappointing that no clear recommendation but four alternative recommendations are given without any guidance which one to select when. That sounds a bit like ‘anything goes’. The reduction from the six approaches found in the LSRs to date to the four recommended is not very impressive. I was also surprised that the recommendations were solely based on the experience of the authors. Why is no attempt made to consult survey methodologists and end-users of LSRs, e.g. by performing a Delphi study? Also Cochrane Methods Groups and the editors of the Cochrane Handbook seem not to have been approached with a request to state their view on the issue.
- The findings presented are part of a larger project on the methods of LSRs about which near to nothing is said in the manuscript. That makes one wonder whether this is not too small a part of the harvest to be optimally useful. Please explain why this element on flow charts is separated from the rest.
- The data set is quite small: 32 LSRs of which 8 are only available as study protocol, 12 have only one (base) version, and 12 have one or more updates. Why is no indication provided of the corresponding imprecision, e.g. by presenting 95% confidence intervals?
- The bottom half of table 2 presents how the study flow is reported among the 12 LSRs that got at least one update. I recommend to do this for all 32 LSRs included, assuming that when no update is yet reported the envisioned handling of flow charts should be specified in either the review protocol or the base version of the review.

Is the work clearly and accurately presented and does it cite the current literature?

Yes

If applicable, is the statistical analysis and its interpretation appropriate?

No

Are all the source data underlying the results available to ensure full reproducibility?

Yes

Is the study design appropriate and is the work technically sound?

Partly

Are the conclusions drawn adequately supported by the results?

Yes

Are sufficient details of methods and analysis provided to allow replication by others?

Yes

Reviewer Expertise:

Methodology, epidemiology, research integrity, open science, systematic review methods.

I confirm that I have read this submission and believe that I have an appropriate level of expertise to confirm that it is of an acceptable scientific standard, however I have significant reservations, as outlined above.

# Response

**Published:** 2022-01-07

**Authors:** Lara Kahale

**Affiliations:** American University of Beirut, Lebanon

The Reviewers’ comments are in **bold** font and our replies in regular font. Extracts from the text are in *italic* fonts with changes *underlined*. We have indicated the sections where revisions have been made in our manuscript.

Reviewer 1: Lex Bouter

**Living Systematic Reviews (LSRs) are updated as new evidence becomes available and gained popularity during the Covid-19 pandemic. This manuscript describes the way PRISMA 2020 flow diagrams are handled in 32 LSRs with a view to recommend how this can best be done. The topic is relevant albeit a bit narrow and the manuscript is written clearly. However, there’re some issues that should to be solved in the next version of the manuscript.**

**Major issues**

**1. It’s disappointing that no clear recommendation but four alternative recommendations are given without any guidance which one to select when. That sounds a bit like ‘anything goes’. The reduction from the six approaches found in the LSRs to date to the four recommended is not very impressive. I was also surprised that the recommendations were solely based on the experience of the authors. Why is no attempt made to consult survey methodologists and end-users of LSRs, e.g. by performing a Delphi study? Also Cochrane Methods Groups and the editors of the Cochrane Handbook seem not to have been approached with a request to state their view on the issue.**

- Thank you for your comment. This study is a methodological survey aiming to summarize what current LSR authors are reporting. Given that the full methodology of living systematic reviews is still emerging, we prefer not to make firm recommendations but lay out options. However, this study is part of a bigger project aiming to develop extension to the PRISMA 2020 statement for LSRs (please see registration form on EQUATOR network website: Equator Network. *PRISMA for LSR – Extension of PRISMA 2020 for living systematic reviews*. 2021; Accessed from https:// www.equator-network.org/library/reporting-guidelines-under-development/reporting-guidelines-under-development-for-systematic-reviews/#LSR] ) . Consistently with the Reviewer’s suggestions, we will be following the EQUATOR Network’s guidance for developing health research reporting guidelines which include, but is not limited to, engaging stakeholders, including methodologists, LSR end-users, Cochrane groups, journal editors, etc. in Delphi exercise. In addition, we will be doing a scoping review of current guidance documents and methods papers, and a qualitative study with stakeholders. We have elaborated about this research under implications for future research.

**2. The findings presented are part of a larger project on the methods of LSRs about which near to nothing is said in the manuscript. That makes one wonder whether this is not too small a part of the harvest to be optimally useful. Please explain why this element on flow charts is separated from the rest.**

- Thank you for your query. This paper is the first published study within the larger project which aims to explore how LSRs authors are currently reporting, conducting, and publishing LSRs. We cite the protocol of that project (Khamis 2019). This study focuses on reporting flow diagrams, another study will focus on general characteristics and delay in updating LSRs, and a third study will focus on methodological features of LSRs. We have now updated our results to include data from up to April 2021, and our dataset now includes 279 publications of 76 LSRs. 

 

**3. The data set is quite small: 32 LSRs of which 8 are only available as study protocol, 12 have only one (base) version, and 12 have one or more updates. Why is no indication provided of the corresponding imprecision, e.g. by presenting 95% confidence intervals?**

- Thank you for your comment. Unfortunately, that was the state of the science. However, as noted in the previous comment, we have now updated our search to include results up to April 2021 Including data from 279 publications of 76 LSRs. We avoided including confidence intervals for the simplicity of presentation and given the small sample size. We are happy to include these if the editor prefers so.

**4. The bottom half of table 2 presents how the study flow is reported among the 12 LSRs that got at least one update. I recommend to do this for all 32 LSRs included, assuming that when no update is yet reported the envisioned handling of flow charts should be specified in either the review protocol or the base version of the review**.

- Thank you for your suggestion. We have checked and found the following (now added to the text): ‘ *None of the 279 living publications reported in their methods section how they plan to report on the study flow’*.
